# Circulating monocytes and tumor-associated macrophages express recombined immunoglobulins in glioblastoma patients

**DOI:** 10.1186/s40169-019-0235-8

**Published:** 2019-06-03

**Authors:** Svenja Busch, Marina Talamini, Steffen Brenner, Amr Abdulazim, Daniel Hänggi, Michael Neumaier, Marcel Seiz-Rosenhagen, Tina Fuchs

**Affiliations:** 1 Institute for Clinical Chemistry, Medical Faculty Mannheim of Heidelberg University, 68167 Mannheim, Germany; 2 Department of Neurosurgery, University Hospital Mannheim, Heidelberg University, 68167 Mannheim, Germany

**Keywords:** Glioblastoma, Immunoglobulins, Monocytes, Tumor-associated macrophages, Tumor volume

## Abstract

**Background:**

Glioblastoma is the most common and malignant brain tumor in adults. Glioblastoma is usually fatal 12–15 months after diagnosis and the current possibilities in therapy are mostly only palliative. Therefore, new forms of diagnosis and therapy are urgently needed. Since tumor-associated macrophages are key players in tumor progression and survival there is large potential in investigating their immunological characteristics in glioblastoma patients. Recent evidence shows the expression of variable immunoglobulins and TCRαβ in subpopulations of monocytes, in vitro polarized macrophages and macrophages in the tumor microenvironment. We set out to investigate the immunoglobulin sequences of circulating monocytes and tumor-associated macrophages from glioblastoma patients to evaluate their potential as novel diagnostic or therapeutic targets.

**Results:**

We routinely find consistent expression of immunoglobulins in tumor-associated macrophages (TAM) and circulating monocytes from all glioblastoma patients analyzed in this study. However, the immunoglobulin repertoires of circulating monocytes and TAM are generally more restricted compared to B cells. Furthermore, the immunoglobulin expression in the macrophage populations negatively correlates with the tumor volume. Interestingly, the comparison of somatic mutations, V-chain usage, CDR3-length and the distribution of used heavy chain genes on the locus of chromosome 14 of the immunoglobulins from myeloid to B cells revealed virtually no differences.

**Conclusions:**

The investigation of the immunoglobulin repertoires from TAM and circulating monocytes in glioblastoma-patients revealed a negative correlation to the tumor volume, which could not be detected in the immunoglobulin repertoires of the patients’ B lymphocytes. Furthermore, the immunoglobulin repertoires of monocytes were more diverse than the repertoires of the macrophages in the tumor microenvironment from the same patients suggesting a tumor-specific immune response which could be advantageous for the use as diagnostic or therapeutic target.

**Electronic supplementary material:**

The online version of this article (10.1186/s40169-019-0235-8) contains supplementary material, which is available to authorized users.

## Background

Macrophages are omnipresent versatile immune cells with myeloid origin. Due to their function as phagocytes they are assigned to the innate immune system where they play a key role in chronic inflammation [1, 2].

Traditionally, the expression of combinatorial immune receptors represented by immunoglobulins (Ig) and T cell receptors (TCR) is thought to be an exclusive competence of lymphoid effector cells like B and T cells [2, 3]. However, in the past decade several publications provided evidence for the recombination of variable immune receptors in cells not belonging to the lymphoid lineage [4, 5]. The initial observations by Puellmann et al. in 2006 demonstrated the expression of variable αβ T cell receptors in a subpopulation of neutrophil granulocytes [6–8]. Ensuing studies showed the recombination of TCR αβ/γδ in monocytes and macrophages [9, 10] as well as TCR γδ in eosinophils [11]. Interestingly, recent studies present the induction of TCRβ expression in neutrophils and macrophages during malaria infection [12, 13]. Furthermore, an implication of these TCR based myeloid variable immune receptors in several chronic diseases like autoimmune disease [14], chronic periodontitis [15], tuberculosis [9] and atherosclerosis [16] was shown. Additionally, our laboratory demonstrated the recombination of variable TCRαβ by macrophages in the tumor microenvironment [17]. Importantly, most recent studies from our laboratory and others provided evidence for the expression of the second variable immune receptor based on immunoglobulin heavy and light chain genes by myeloid immune effector cells [18–20].

Traditionally, macrophages are divided into M1 and M2 categories. In the tumor milieu, M1 macrophages are typically tumor-suppressive and M2 macrophages serve an anti-inflammatory, tumor-supportive role [21]. The tumor-associated macrophages (TAM) play a key role in the host’s immune response to the tumor. Typically, TAM are attributed to promote tumor growth and progression in two different ways, directly by stimulating tumor cell proliferation and indirectly by creating an immunosuppressive microenvironment [22–26]. Moreover their infiltration is associated with a poor clinical outcome in cancer patients [27, 28]. But despite all these findings, the exact role of tumor-associated macrophages is still controversial.

Glioblastoma multiforme (GBM) is the most common malignant tumor of the central nervous system in adults with a global incidence of 0.59–3.69/100,000 [29]. It is associated with poor prognosis and a median patient survival of 12–15 months from diagnosis [30, 31]. The long-term survival for glioblastoma patients, which is considered to be more than 3 years from diagnosis, is only at around 3–5% [32, 33]. Since GBM is rapidly fatal, therapy includes surgical resection to the extent feasible, adjuvant radiotherapy and temozolomide chemotherapy and is mostly only palliative [30, 34, 35].

Future prospects in the treatment of glioblastoma tend to immunotherapies or targeting of the tumor microenvironment. As the exact immunological features of TAM in glioblastoma is still unknown, we investigated the immunoglobulin repertoires of monocytes/macrophages in the tumor tissue and peripheral blood of 17 glioblastoma patients and compared them to the immunoglobulin repertoires of corresponding B cells. Detailed analyses of the immunoglobulin sequences are needed as these myeloid immunoglobulins might represent novel biological targets, since immunoglobulin variants are discussed as potential biomarkers and therapeutics in cancer [36].

In summary, the goal of this study is to determine whether patients with glioblastoma have a specific repertoire of myeloid antibodies, if there are differences in the immunoglobulin repertoires of circulating monocytes and tumor-associated macrophages from glioblastoma patients and if the myeloid antibodies can be a useful tool as therapeutic or diagnostic target in glioblastoma.

## Materials and methods

### Patient cohort

All 17 patients included in the study suffered from glioblastoma, which was assigned to WHO grade IV. After prior written consent, tumor tissue and peripheral blood for study purposes were obtained during surgery. The average age of the patients was 58 years with an age range from 43 to 74 years. An overview of the patient’s characteristics is listed in Additional file 1: Table S1.

### Cell isolation from tumor tissue/blood and purity control

Both the tissue and the blood samples were processed immediately after collection in the operating room. The fresh tumor tissue was manually divided into small pieces with a sterile single-use scalpel and enzymatically digested with a solution of DNase I (500 U/ml) and collagenase IV (190 U/ml) in RPMI at 37 °C and 5% CO_2_ on a rotator for at least 90 min. Finally, the cell suspension was filtered and washed with PBS.

The blood sample was diluted 1:1 with PBS (Sigma Aldrich) and separated by density gradient centrifugation using Ficoll-Paque PLUS solution (GE Healthcare Life Sciences). The white layer of PBMCs was transferred to a fresh tube and washed with PBS. If necessary, one or two erythrocyte lysis steps followed. Subsequently, the cell suspensions from tumor and blood sample were subjected to CD14 and CD19 Magnetic Cell Sorting (MACS, Miltenyi Biotec) to isolate monocytes/macrophages as well as B cells. The purified cell fractions were resuspended in 1 ml of TRIzol LS Reagent, respectively (Thermo Fisher). The purity of the isolated cell fractions was either tested by flow cytometry or the absence of B and T cells was demonstrated by using a highly sensitive PCR protocol.

### Repertoire-PCRs of immunoglobulins

For the analysis of gene expression, RNA was isolated according to the TRIzol LS Reagent manual by Thermo Fisher. cDNA synthesis was carried out using equal amounts of RNA to get comparable results in the following PCR. For every isolated cell fraction a quality control PCR was conducted using primers for GAPDH, CD2, CD14, CD19, CD68 and CD163. Repertoire-PCRs for the desired immunoglobulins were performed for all cell fractions which passed the quality control. Depending on the studied antibody class, one reverse primer in the constant region of the specific immunoglobulin sequence and four to seven different consensus forward primers in the variable regions were used. In total, four antibody classes (IgM, IgG, Igκ, Igλ) were investigated. The sequences of the primers used in this study can be provided upon request.

### Cloning and Sanger sequencing

For the generation of the immunoglobulin sequences, the PCR products were cloned into competent *E. coli* (Top10) by using the TOPO TA Cloning Kit for Sequencing (Thermo Fisher) according to the manufacturer’s protocol. The generated plasmids were purified out of liquid cultures using the GeneJET Plasmid Miniprep Kit (Thermo Fisher). Sequencing PCR was performed using the BigDye Terminator v1.1 Cycle Sequencing Kit (Thermo Fisher). The amplified sequencing-PCR-products were purified with the NucleoSEQ kit from Macherey–Nagel and then used for Sanger sequencing on an ABI Prism 310.

### Sequence analysis

For the analyses of the sequencing results the databases igBLAST and VBASE2 were used. All alignments, mutation analyses and the construction of an own database were done using CLC Genomics Workbench by Qiagen.

### Immunohistochemistry

CD14 immunostaining and HE staining of glioblastoma tissue was performed using standard protocols in the department of neuropathology of the university hospital Heidelberg. Analysis of the staining was conducted on an Olympus microscope IX70 with the camera “Progres Gryphax” by Jenoptik.

### Cytokine release assay

The cytokine levels in the patient’s serum were analyzed using the Milliplex MAP Human Cytokine/Chemokine Magnetic Bead Panel (Merck Millipore). The patient samples and seven healthy controls were collected during the study and stored at − 20 °C until the last patient was included. The assay was performed according the manufacturer’s protocol with the standards and quality controls in duplicates and all samples in triplicates. The plate was analyzed using the Milliplex Analyzer (Merck Millipore).

### Quantitative volumetry of the tumors

All brain tumor volume measurements were performed using the iPlan Cranial Software 3.0.5 (Brainlab AG).

### Statistical analysis

The student’s t-test was used to compare the significance of repertoire diversity, mutation rate and cytokine release between the different cell groups. *p > 0.05 and **p < 0.001 was considered statistically significant. The Shannon diversity index is a mathematical tool to estimate variability of the immunoglobulin repertoires. It is expected that monoclonal and oligoclonal samples have low values, and highly diverse samples result in higher values [37, 38].

## Results

### High purity of the monocyte/macrophage cell preparations

**Fig. 1:**
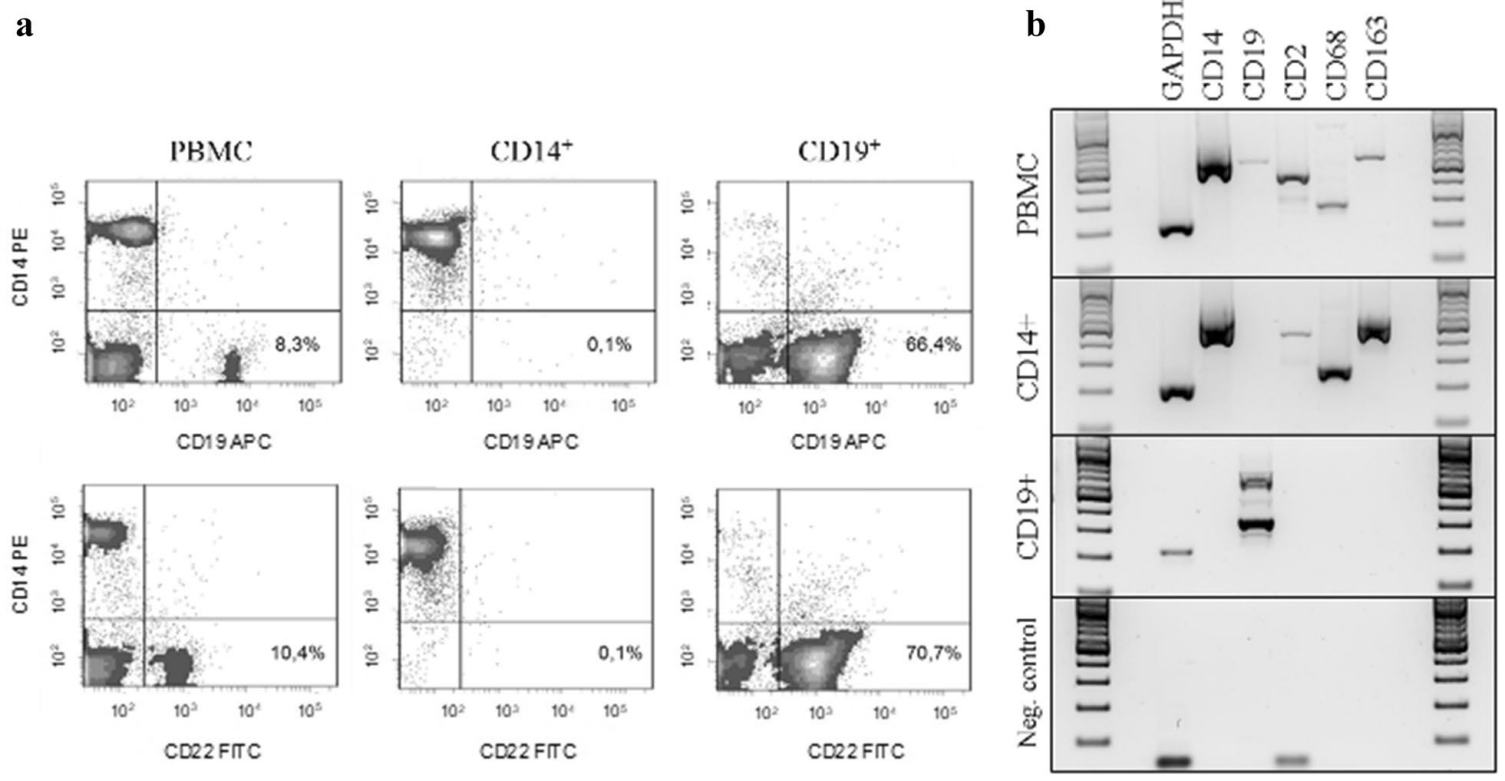
Purity of the isolated CD14-positive cells validated by flow cytometry and gene expression profiling. **a** Flow cytometry of freshly isolated MACS purified CD14^+^ monocytes of one representatively chosen blood sample demonstrating purity of routinely 99.9% and absence of CD19^+^/CD22^+^ B cells. The analyses of PBMCs and CD19^+^ B cells are shown as positive controls for the used antibodies. **b** RT-PCR profiling is shown for CD14^+^ monocytes from one representative healthy individual. Expression of GAPDH, B cell (CD19), T cell (CD2) and monocyte/macrophage marker genes (CD14, CD68, CD163) demonstrates the quality of the isolated cell preparations and the absence of detectable quantities of B or T cells. PBMC and CD19^+^ B cells are demonstrated as reference. *GAPDH* glycerinaldehyd-3-phosphat-dehydrogenase, *PBMC* peripheral blood mononuclear cells

Recent evidence revealed the expression of immunoglobulins from macrophage subpopulations in the tumor microenvironment [20]. In this proof-of-principle study, different tumor entities were tested for the presence of the immunoglobulin expressing TAM subpopulation including one patient with glioblastoma. Here, we systematically investigated immunoglobulin repertoires of TAM from 17 glioblastoma patients (Additional file 1: Table S1) and compared them to the circulating monocytes of the same patients. To address this, we first established a protocol for the reproducible isolation of CD14^+^ cells from venous blood and tumor tissue. The purity of the isolated monocyte/macrophage populations was tested by flow cytometry. As these experiments showed a purity of > 99% the quality of the isolation method was confirmed (Fig. 1a). Using a highly sensitive quality control PCR protocol the isolated cell fractions routinely showed no characteristic gene expression of B and T cell markers (Fig. 1b, Additional file 1: Figure S1). Furthermore, the presence of the myeloid markers CD14, CD68 and CD163 confirmed the isolated cells to be monocytes/macrophages (Fig. 1b). Consequently, the highly pure isolated cell fractions were used for further analysis of the immunoglobulin expression. Of note, expression of the secreted and membrane form of IgG could be identified in the monocyte/macrophage cell preparations from healthy individuals and glioblastoma patients (Additional file 1: Figure S2A).

### Higher immunoglobulin diversity in circulating monocytes than in the tumor-associated macrophages

We analyzed the CDR3 heavy and light chain Ig repertoires expressed by TAM and monocytes obtained from 15 patients with glioblastoma during surgery. In order to do this, the IgM, IgG, Igκ and Igλ repertoire diversities were determined by cloning and Sanger sequencing of the expressed Ig CDR3 variants. Peripheral blood B cells and B cells isolated form the tumor from three representative glioblastoma patients (GBM003; GBM004; GBM008) were co-analyzed as reference. In total, 1293 immunoglobulin sequences coding for 456 unique CDR3 sequences were included in this study.

**Fig. 2:**
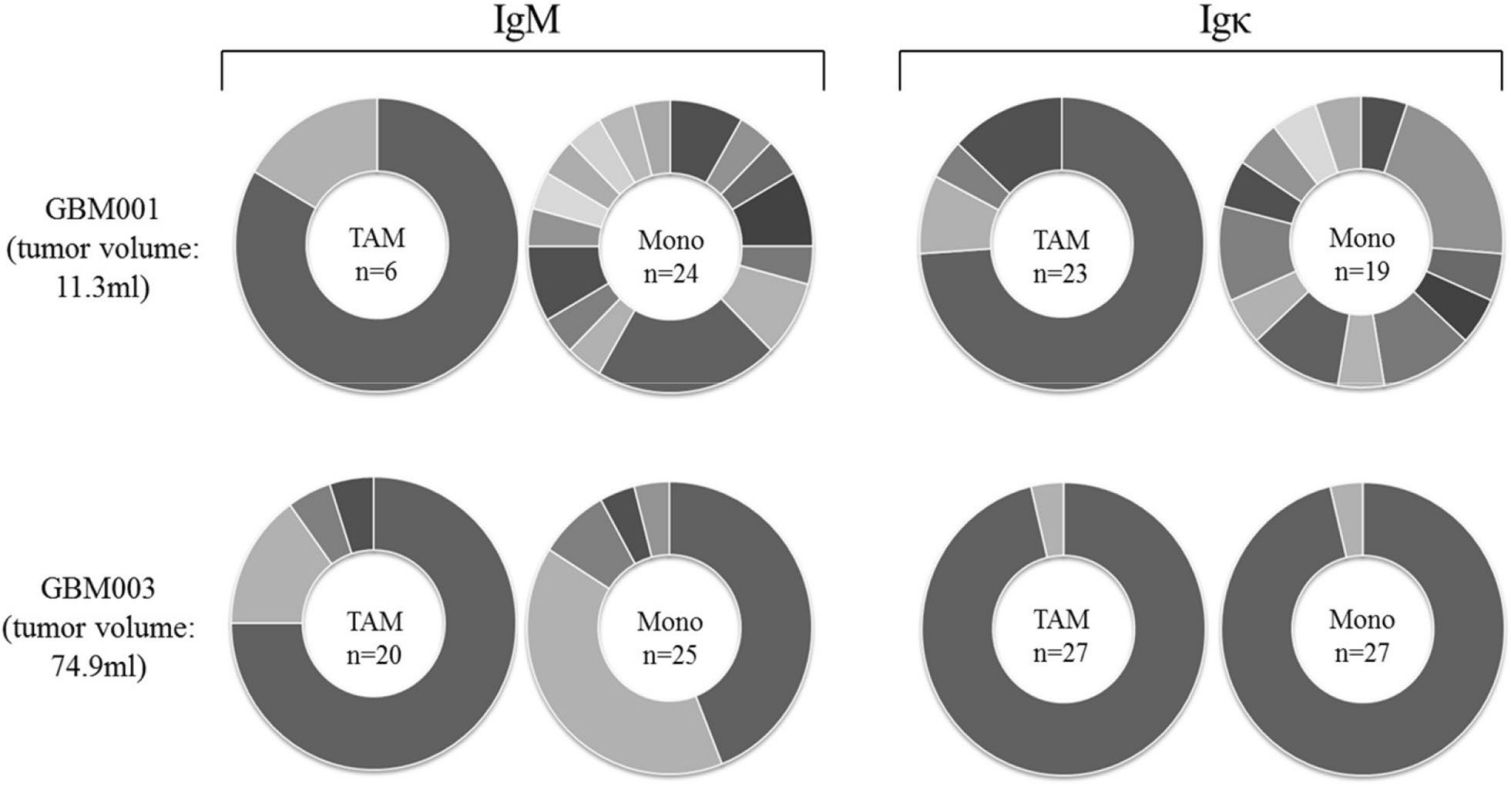
TAM Ig heavy and light chain repertoires are less diverse than those of circulating monocytes. IgM and Igκ repertoire diversities in circulating monocytes and tumor macrophages from two representative patients (GBM001 and GBM003). Sector areas correspond to the relative frequency of individual clonotypes within a given Ig CDR3 repertoire and are sorted by size. Each sector within a circle chart represents a unique CDR3 variant and the total number of identified variants is shown in the center. Note that TAM express less diverse IgM and Igκ repertoires than monocytes from the same patients. The tumor volumes of the two patients are shown (11.3 ml and 74.9 mL, respectively). The repertoire diversities of all analyzed immunoglobulin sequences from all patients in this study are shown in (Additional file 1: Figure S4)

Hence, analysis of the immunoglobulin expression revealed diverse immunoglobulin heavy and light chain repertoires of monocytes and tumor-associated macrophages in all glioblastoma patients. Interestingly, the immunoglobulin diversity between monocytes and B cells from the blood and macrophages isolated from the corresponding tumor sample of the same patient differed strongly (Additional file 1: Figure S3A). In all patients, the diversity of the expressed immunoglobulins in the circulating monocytes was much higher than in the corresponding tumor-associated macrophages (Fig. 2, Additional file 1: Figure S4). Furthermore, the immunoglobulin expression level of circulating monocytes was usually higher than in TAM (Additional file 1: Figure S2B). These results were also reflected by the Shannon Diversity Indices 0.95 for monocytes and 0.42 for TAM (Additional file 1: Figure S3B). The size of the immunoglobulin repertoires from the circulating monocytes as well as the tumor-associated macrophages of different patients was individual specific. Large variances from eighteen different immunoglobulin heavy chain clonotypes in the circulating monocytes and only two different clonotypes in the tumor-associated macrophages from one patient were found. A similar phenomenon could be identified in the immunoglobulin light chain repertoires (Fig. 2, Additional file 1: Figure S4).

### Inter- and intra-individual overlaps of immunoglobulin CDR3 variants

Most of the immunoglobulin CDR3 nucleotide sequences from TAM and monocytes (97.8% of all immunoglobulin heavy and light chain CDR3 variants) were found in only one subject. However, five immunoglobulin heavy chain CDR3-regions were found that were expressed in more than one patient (Table 1). In the B cell preparations from three representative GBM patients no CDR3 heavy chain sequences were shared between the different patients. However, we found two immunoglobulin heavy chain sequences in the B cell preparations shared between blood and tumor of the same patient (Additional file 1: Table S2). In the monocytic cells, we found seven immunoglobulin heavy chain sequences in blood and tumor of the same patient. The immunoglobulin light chain sequences showed slightly more overlapping sequences in monocytes and TAM (ten CDR3 variants) as well as in the B cells (four CDR3 variants) (Table 1 and Additional file 1: Table S2). Summarizing, most of the myeloid immunoglobulin heavy and light chain sequences are individual specific but show a higher percentage of shared CDR3 variants than the B lymphocytic cells.

**Table 1:** Shared immunoglobulin heavy and light chain CDR3 variants in monocytes and TAM from 15 glioblastoma patients

CDR3	GBM001		GBM002		GBM003		GBM004		GBM005		GBM006		GBM0007		GBM008		GBM009		GBM010		GBM011		GBM012		GBM013		GBM014		GBM015	
	TAM	Blood	TAM	Blood	TAM	Blood	TAM	Blood	TAM	Blood	TAM	Blood	TAM	Blood	TAM	Blood	TAM	Blood	TAM	Blood	TAM	Blood	TAM	Blood	TAM	Blood	TAM	Blood	TAM	Blood
*Shared immunoglobulin heavy chains*																														
VRSWAYSLTPAGTGTYYFES		X	X																											
GRGGTGYYYYMDV			X	X																										
ARGAGVRYFDWLSPAGY			X		X	X																								
ARVPINYDILTGTDY					X	X																								
ARDEKQLVRNYYYYYGMDV																X	X							X						
AKTLMVRGVRNAFDI																							X		X	X				
AGEGSVVLTTSTFDI																							X	X						X
ARDWRSGGSCYYY																							X	X						
*Shared immunoglobulin light chains*																														
HQSSNLPWT	X			X																										
IRVVIYLGR	X			X									X	X		X			X			X								
MQGTHWPPTWT	X	X		X			X								X															
QQYYRIPCT		X	X																											
QHYNNWPWT									X											X		X								
QHYNDWPWT									X									X												
QHYNAWPPWT										X								X												
NTIMPGLRGR										X								X												
QQSYSTPLT																X										X				
QAWDSSTAV																											X	X		

Shown are the shared immunoglobulin heavy (n = 8) and light chain (n = 10) CDR3 variants of monocytes and TAM from 15 glioblastoma patients. The CDR3 sequences were marked with an “X” in the respective cell preparations. Shared CDR3 variants were found between different glioblastoma patients (inter-individual sharing) and between the monocyte and TAM preparations of single glioblastoma patients (intra-individual sharing)

### Diversity of the immunoglobulin repertoire from the tumor-associated macrophage inversely correlates with the tumor volume

The diversity of the immunoglobulin repertoires in TAM and monocytes varied strongly between the individual patients. The patient characteristics (tumor stage, IDH mutation, ARTX mutation, etc.) of the glioblastoma patients included in our study were quite uniform (Additional file 1: Table S1). However, one characteristic distinction between the single patients is their unique tumor volume which ranged from 3 to 92 ml.

**Fig. 3:**
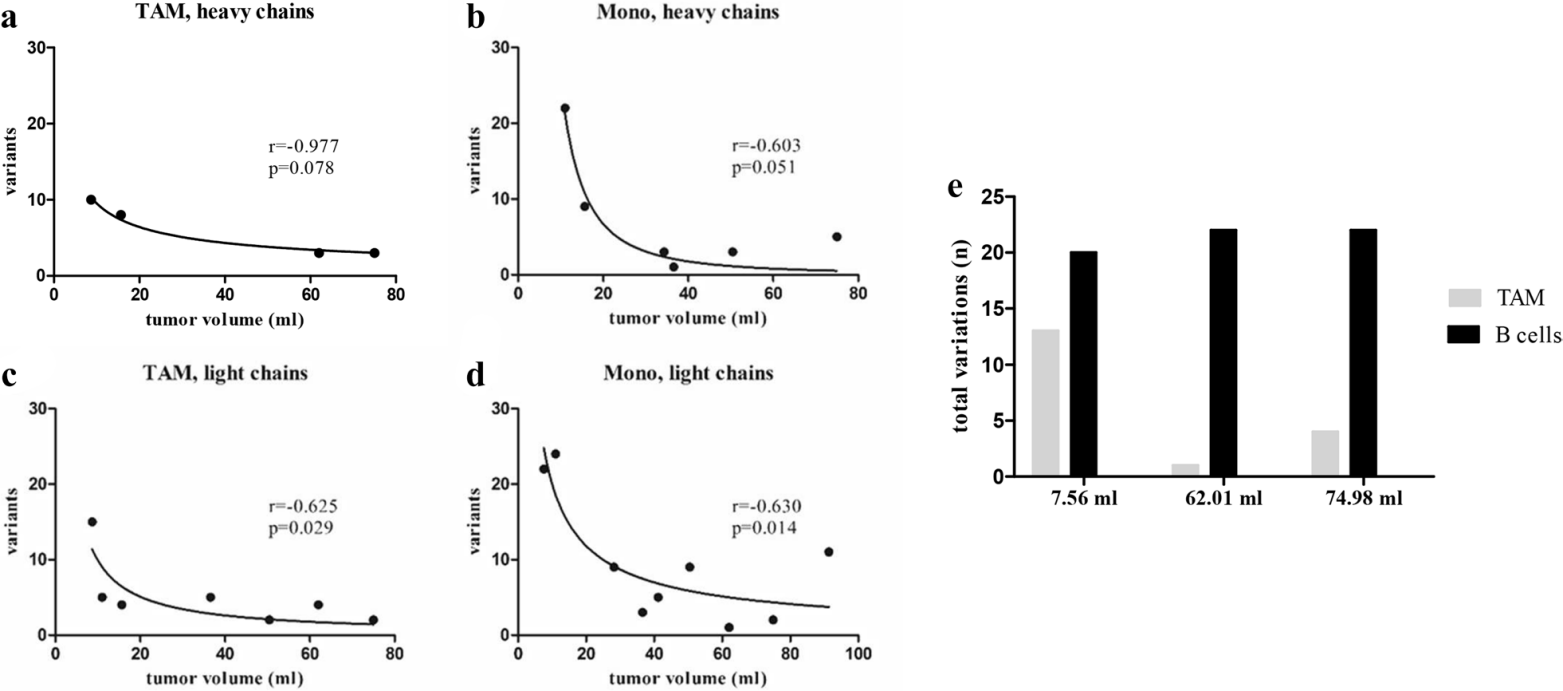
Inverse correlation between the diversity of the immunoglobulin-repertoire and the tumor volume. The total number of different immunoglobulin CDR3 variants from IgM- and IgG-heavy chains expressed in tumor-associated macrophages (**a**) and circulating monocytes (**b**) from glioblastoma patients is negatively correlated to the corresponding tumor volume. The inverse correlation of the total number of different CDR3 variants in tumor-associated macrophages (**c**) and circulating monocytes (**d**) and the tumor volume was confirmed in the immunoglobulin light chain repertoires (Igκ- and Igλ). For all graphs (**a**–**d**) the respective correlation coefficients r and the p-values are indicated. **e** The total number of different IgM and IgG CDR3 variants from tumor-associated macrophages and B cells of three patients are shown. The immunoglobulin repertoires expressed by TAM show a higher repertoire diversity in the small tumor than in the large tumors. In contrast, the corresponding B cells of the same patients show a high repertoire diversity independent of the tumor volume

Surprisingly, the diversities of the expressed immunoglobulin repertoires in TAM and monocytes, respectively, decrease with increasing tumor volume (Fig. 3a–d). The decrease of diversity with increasing tumor volume was more pronounced in monocytes. The inverse correlation is indicated by correlation coefficients between − 0.603 and − 0.977. Especially the light chains show a strong significant correlation with p = 0.029 for TAM and p = 0.014 for monocytes (Fig. 3c, d). Importantly, the number of CD14^+^ cells isolated from the tumor and blood samples did not correlate with the tumor volume (Additional file 1: Figure S5), and histological analysis revealed an infiltration of a diverse number of CD14^+^ cells in tumors of different sizes (Additional file 1: Figure S6). Of note, other patient characteristics showed no correlation with the immunoglobulin CDR3 repertoire of monocytes and TAM (data not shown).

Contrasting to the immunoglobulin repertoire from macrophages, the immunoglobulin diversity from B cells showed no correlation with the tumor size (Fig. 3e).

### Immunoglobulin repertoire features of the CDR3 sequences from myeloid cells and B cells

**Fig. 4:**
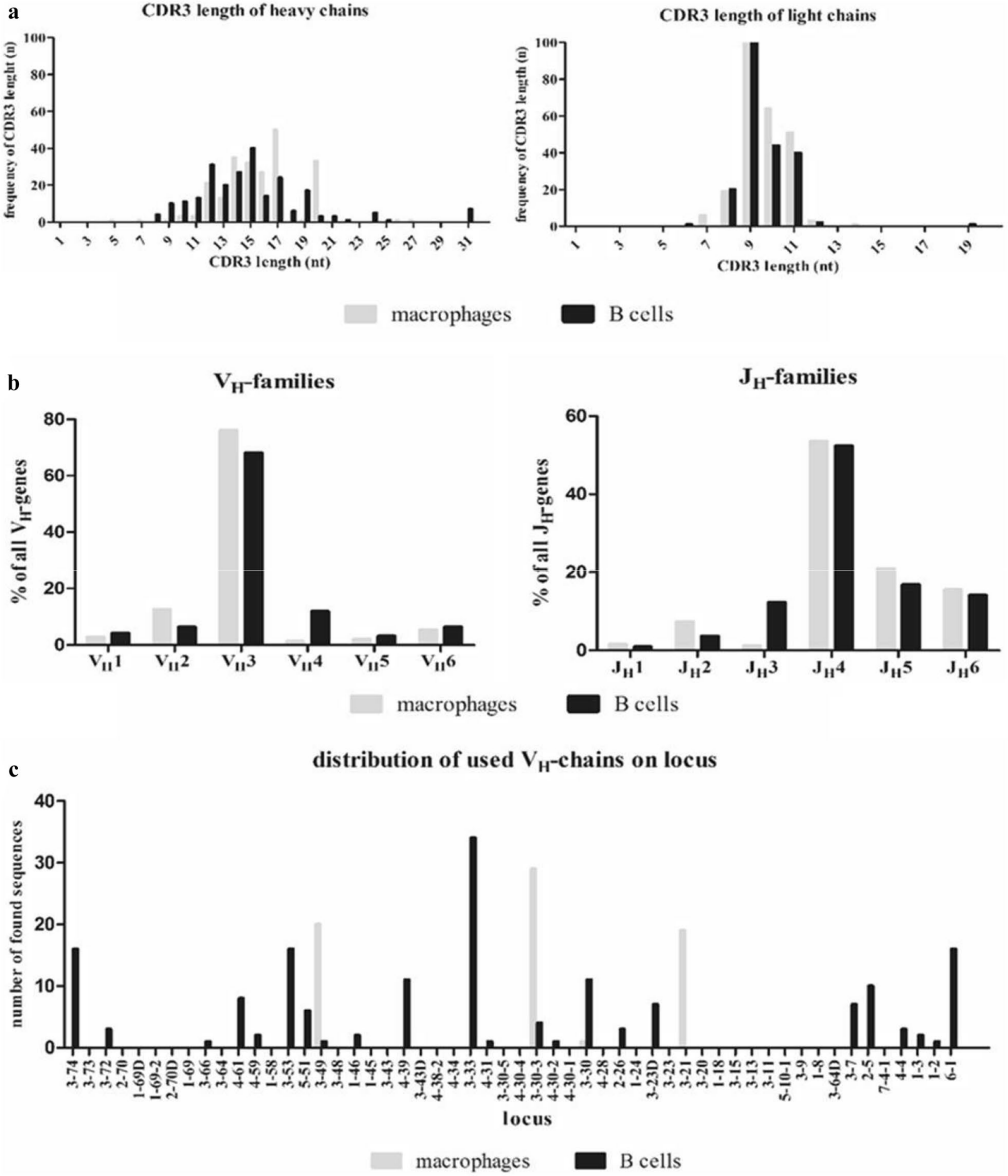
Immunoglobulin repertoire features of macrophages and B cells. **a** The immunoglobulin heavy (left) and light chain (right) CDR3-length distributions are shown for the investigated tumor-associated macrophages and B cells from three representative patients. The number of nucleotides of the respective CDR3 sequence length (nt, x-axis) is plotted against the frequency of the respective CDR3 sequence length (n, y-axis). n = total number; nt = nucleotide. **b** The V_H_- and J_H_-family gene usage by TAM and B cells are indicated. The frequency (in %) of V_H_ family genes (left) and J_H_-family genes (right) expressed by TAM and B cells, respectively, from three representative glioblastoma patients demonstrate a similar genes usages in both cell groups. **c** Distribution of the expressed V-chain genes of tumor-associated macrophages and B cells from three representative glioblastoma patients on the immunoglobulin heavy chain locus of chromosome 14. Here, macrophages show a restricted distribution with focus in the center of the locus compared to B cells which express more widely distributed V-chains. The distribution of all V chain genes expressed by TAM analyzed in this study is shown in Additional file 1: Figure S7

A detailed analysis of the myeloid and B cell immunoglobulin repertoires was performed to investigate if the differences in immunoglobulin repertoire size between the cell populations are based on sequence specificities. Therefore, reference alignment and structural analysis of the immunoglobulin sequences of TAM and B cells from three representative patients provided detailed sequence information, such as the exact V gene and J gene usage and CDR3 length. Analysis of the immunoglobulin heavy and light chain CDR3 lengths from TAM and B cells varied only slightly between TAM and B cells (Fig. 4a). As expected, the CDR3 sequences originating from B cells demonstrated Gaussian-type profiles. In contrast, the CDR3 length distribution in TAM showed a more oligoclonal profile in the three patients analyzed.

Additionally, the distribution of the sequenced V_H_- and J_H_-families was investigated. For this purpose, the expressed IgM and IgG heavy chains of all glioblastoma-patients were summarized, grouped into families and compared to the corresponding B cells (Fig. 4b). Most of the expressed V_H_-segments from tumor-associated macrophages and B cells (76% and 68%, respectively) belong to the V_H_3-family while the rest is equally distributed among the other five families. The most used J_H_-chains are J_H_4 with 53% in macrophages and 52% in B cells followed by J_H_5 (21% and 17%) and J_H_6 (15% and 14%), respectively. This is in accordance with previously published numbers for normal B cells [39].

Thus, based on this analysis, we found that the distributions of immunoglobulin V_H_- and J_H_-family genes were comparable between B cells and TAM. Of note, the three most expressed V_H_-genes in monocytes and TAM are 3-30-3*01 (64×), 3-21*01 (39×) and 3-33*01 (32×) whereas B cells expressed predominantly 3-33*01 (22×), 6-1*01 (13×) and 3-11*01 (12×).

### Analysis of the chromosomal localization of V_H_-region genes

Next, to assess whether the restriction of the immunoglobulin repertoires in TAM had its origin in the localization of the V_H_ region genes on the chromosome, V_H_ region genes were mapped regarding to their chromosomal location for a comparative overview. The V_H_ region genes of the Ig heavy chains are located at the telomeric end of chromosomal band 14q32.33.

The immunoglobulin repertoires of macrophages and B cells from three representative glioblastoma patients with a small, medium and large tumor were analyzed (GBM003, GBM004, GBM008; Fig. 4c). Here, V_H_ region genes expressed by B cells were widely distributed over the entire locus with different levels of expression as expected. In contrast, the V_H_ region genes expressed by TAM of the same patients were clustered in the center of the locus. However, the clustering was less pronounced when immunoglobulin V_H_ region genes expressed by TAM and monocytes from all GBM patients analyzed in this study were taken into account (Additional file 1: Figure S7).

### Similar mutation frequencies of immunoglobulin V_H_-Region genes expressed by tumor-associated macrophages and B cells

**Fig. 5:**
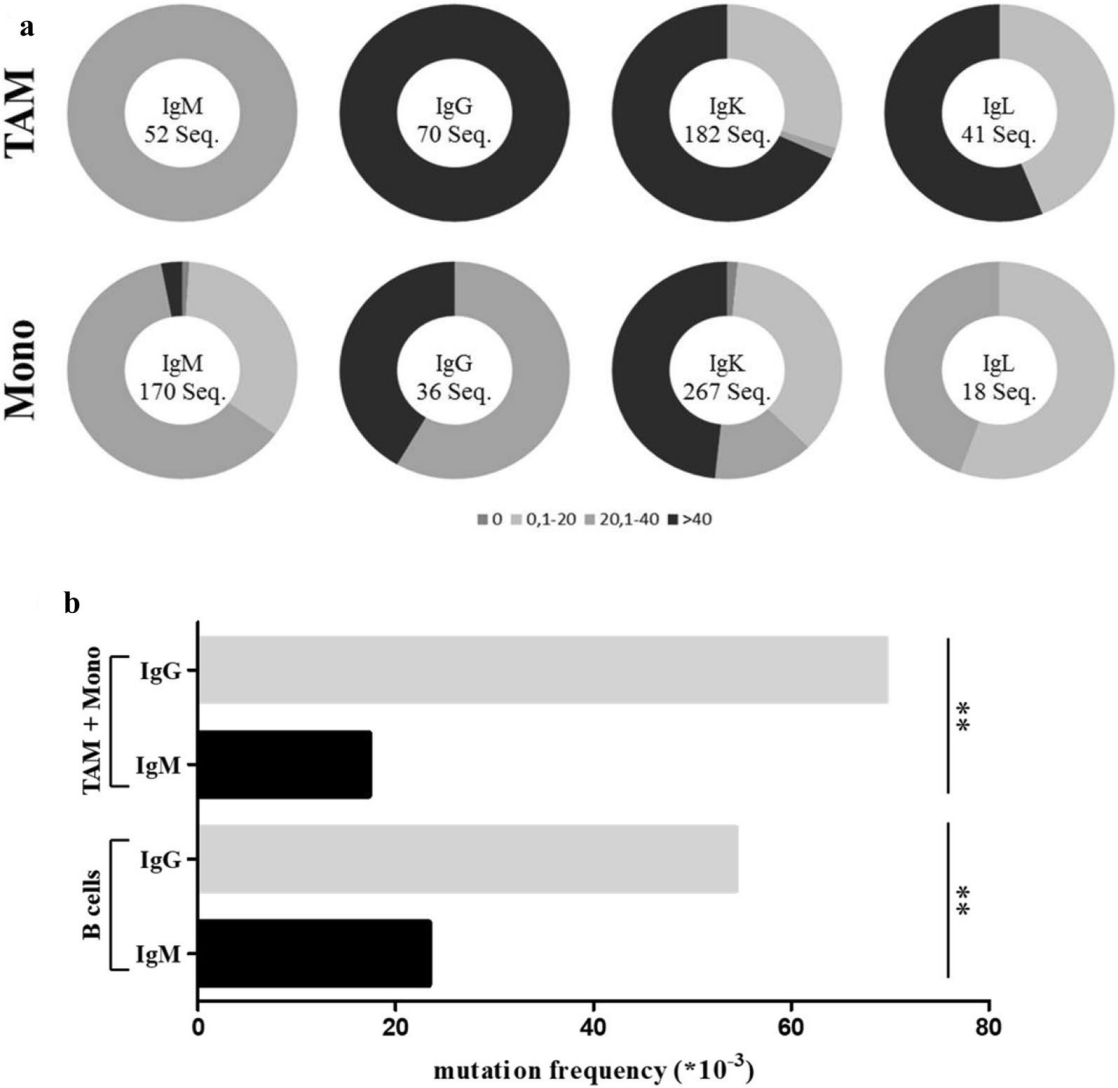
Mutation frequencies in tumor-associated macrophages and circulating monocytes. **a** The mutation frequencies (× 10^−3^) of the heavy chains IgM and IgG as well as the light chains Igκ and Igλ of tumor-associated macrophages and circulating monocytes are demonstrated. For all four Ig classes higher mutation rates were found in TAM than in circulating monocytes. The mutation frequencies were clustered in different categories depending on the number of mutations per V_H_ region gene (0; 0.1–20; 20.1–40; > 40). **b** The overall mutation frequencies of IgM and IgG heavy chains in monocytes/macrophages and B cells are shown. The investigated tumor-associated macrophages and circulating monocytes show the same distribution of mutations as their corresponding B cells. In all investigated cell types, the mutation frequency for IgG is significantly higher (p > 0.01) than for IgM

Functional antibodies are assembled by V-(d)-J gene rearrangement and then diversified by somatic hypermutation. The restricted immunoglobulin repertoire diversities in monocytes/macrophages compared to B cells prompted us to analyze the mutation frequencies of immunoglobulin V_H_-region genes in macrophages and B cells. Therefore, the number of somatic mutations in macrophages as well as in B cells was quantified to examine if a lower number of somatic mutations provided an explanation for the smaller repertoire sizes. Interestingly, comparing the mutation frequencies of immunoglobulin V_H_ region genes from TAM and the corresponding circulating monocytes revealed a higher mutation frequency in TAM than in monocytes although they have a less diverse repertoire. This could be demonstrated for immunoglobulin heavy and light chain V region genes, respectively (Fig. 5a).

Of note, the overall number of mutations in the V_H_ region genes of IgM and IgG from macrophages and B cells showed a similar distribution for both cell types (Fig. 5b). But in general, the number of somatic mutations in IgG sequences was significantly higher than in IgM sequences.

Thus, the differences in the immunoglobulin repertoire diversities between macrophages and B cells are not based on distinct abilities for somatic hypermutation.

### Impaired immunocompetence of monocytic cells from GBM patients

**Fig. 6:**
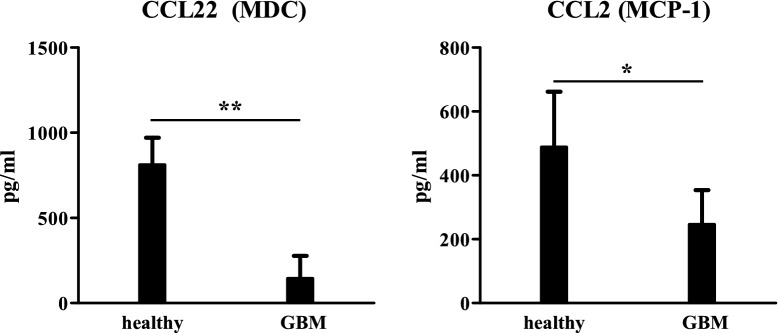
Serum levels of cytokines in healthy individuals and GBM patients. Serum levels of CCL22 (MDC, left) and CCL2 (MCP-1, right) from healthy controls (n = 7) and GBM patients (n = 11) are shown. The cytokine concentrations were measured from serum samples with an multiplex Luminex assays in triplicates. *p < 0.05; **p < 0.001. The serum levels of additional cytokines are shown in Additional file 1: Figure S8

GBM is described to be associated with systemic immune suppression [40]. In this regard, the immune status of the GBM patients was evaluated on the basis of cytokine release in the patients serum, HLA-DR expression of myeloid cells and hematological cell counts. The immune status was analyzed to examine if the restricted immunoglobulin repertoires might be based on impaired immunocompetence of the myeloid cell compartment. To test the overall immune status of the GBM patients in this study, serum cytokine levels of the GBM patients were compared to healthy controls. A number of pro-inflammatory cytokines/chemokines including CCL2 (MCP-1), CCL22 (MDC), sCD40L, CXCL10 (IP-10), CXCL8 (IL-8), EGF and CCL3 (MIP-1α) were (in some cases significantly) decreased in GBM patients compared to healthy controls suggesting a lowered immune competence in GBM patients (Fig. 6, Additional file 1: Figure S8A). Most of these cytokines are primarily produced by monocytes/macrophages and may represent an impaired immune status of these myeloid cells. Interestingly, the anti-inflammatory cytokine IL-10 is increased in the glioblastoma patients which has already been reported in association with immunosuppression in glioblastoma [41], whereas TNFα serum levels were unaltered (Additional file 1: Figure S8B).

Hematological analyses in all patients revealed on average slightly elevated white blood cell counts with decreased lymphocyte, elevated neutrophil and normal monocyte cell counts (data not shown). These conditions have been previously described in glioblastoma patients [42].

HLA-DRA expression in monocytes has been described as a marker for immune competence. Especially in septic patients but also in the context of cancer, low HLA-DRA levels have been reported [40, 43]. A tendency to lower HLA-DRA expression in monocytic cells especially of TAM from GBM patients in this study compared to expression levels in monocytes from healthy individuals are demonstrated (Additional file 1: Figure S9). Consequently, several evidences for impaired immunocompetence of immune cells especially monocytes in the glioblastoma patients are detected and which are not based on decreased numbers of monocytes in the peripheral blood.

## Discussion

Glioblastoma multiforme is the most common brain tumor in adults that is characterized by a marked heterogeneity at the cellular and molecular level [44]. In GBM, tumor-infiltrating microglia and peripheral macrophages are major immune cell populations within the tumor accounting for up to 30% of tumor mass in human GBMs [45]. In a recent study, we presented the expression of recombined immunoglobulin heavy and light chains in tumor-associated macrophages isolated from various types of tumor tissue [20]. In the present study, we were able to show, for the first time, the constitutive expression of immunoglobulin heavy and light chains in tumor-associated macrophages and circulating monocytes in a cohort of glioblastoma patients. Of note, the soluble and the transmembrane forms of immunoglobulins were demonstrated in monocytes and macrophages.

Importantly, highly diverse and individual-specific immunoglobulin heavy and light chain repertoires were detected in the circulating monocytes and in macrophages from tumor tissue of all 15 glioblastoma patients analyzed in this study. However, the immunoglobulin repertoire diversity in the myeloid cells was highly restricted compared to the B cells of three representative patients. This is in accordance with previous reports about restricted variable immunoglobulin and T cell receptor repertoire expression in myeloid cells [9, 17, 18, 20]. Of note, all sequences in this study were generated by cloning and Sanger sequencing of PCR products generated with consensus primers. A primer bias could be ruled out due to the significant differences of the expressed V_H_ genes between the myeloid and B cell populations. Moreover, the number of somatic mutations within the immunoglobulin sequences, the chromosomal localization of the expressed V_H_-genes, the CDR3 length, and the use of V_H_ and J_H_ chain-family genes did not show significant differences between monocytes/macrophages and the B lymphocytic cells. This suggests a pathway of recombination of the individual immunoglobulin gene segments in the myeloid cells which is very similar or even identical to the mechanism used in B cells.

In addition to the lower immunoglobulin diversity in TAM and monocytes compared to B cells, we were able to demonstrate an inverse correlation of the immunoglobulin repertoires with the respective individual tumor size of the GBM patients. For the investigated light chains this inverse correlation was statistically significant (p = 0.029 and p = 0.014), while the significance level of 5% was missed for the heavy chains in both cases. In the future, by extending the patient population and using a high-throughput sequencing approach, however, statistical significance will probably be achieved. The inverse correlation was specific for the myeloid cell compartment and could not be detected in B cells. In the tumor microenvironment, infiltrating macrophages have been described as antitumor M1 as well as protumoral M2 phenotypes [23]. These functional phenotypes are defined by differential expression of surface markers, secreted cytokines, and roles in immunoregulation [45]. However, clinical and mouse model data correlate the accumulation of macrophages with protumoral activities [22–25]. Due to the negative dependence of antibody diversity and tumor size, an immunosuppressive effect of the tumor and its microenvironment on the immunoglobulin-expressing macrophages and monocytes might be conceivable. Larger tumors seem to have stronger suppressive effects on the immunoglobulin expression of myeloid cells than smaller tumors. Importantly, the suppressive effect seems to be more pronounced within the tumor than in the periphery. The observed lowered cytokine release and lowered HLA-DR expression of monocytic cells in the GBM patients are in line with this hypothesis. Impaired immunocompetence on the level of cytokine release and other features have been reported in several studies of glioblastoma [46, 47].

On the other hand, an alternative explanation for our results showing even more restricted immunoglobulin repertoires in the TAM compartment than in the circulating monocytes might be conceivable. Similar results were obtained in studies where variable immune receptors of tumor-infiltrating lymphocytes (TIL) were compared to circulating lymphocytes [48, 49]. These studies showed a clonal restriction due to oligoclonal expansion in the TIL compartment suggesting an adaptation to- or a modification by the tumor microenvironment. Mohme et al. conclude that TIL have undergone antigen-induced activation and should be able to mount a cytotoxic anti-tumor response [49]. Correspondingly, due to the limited immunoglobulin diversity in TAM a tumor-specific selection of TAM leading to specific immunoglobulin expression could be reasonable. In line with this is the high frequency of somatic mutations in the variable regions which indicates that the tumor-infiltrating macrophages locally produce restricted immunoglobulin repertoires with evidence of antigen-driven maturation. It might therefore be possible that the immunoglobulins expressed by TAM are implicated in the anti-tumor immune response. Conceivably, the restricted antigen-specific immunoglobulin variants expressed by the monocytic lineage could be a useful as diagnostic or therapeutic target. Indicative for this is that a more detailed analysis of the macrophage immunoglobulin repertoires revealed that the expressed immunoglobulin CDR3 variants show intra-individual overlaps between the peripheral blood and tumor compartment, as well as inter-individual overlaps of the repertoires between several patients. Interestingly, in almost half the patients we find shared immunoglobulin CDR3 variants between circulating monocytes and tumor-associated macrophages isolated from the same patients suggesting a migration of monocytes expressing specific CDR3 variants to the tumor microenvironment and selection in situ. This is in accordance with a current study showing that 85% of the TAM population in glioma are comprised of infiltrating monocytes and macrophages [50]. It might be conceivable that even more patients show shared CDR3 variants between the blood and tumor compartment with a more sensitive NGS-based repertoire analysis method. To analyze this aspect in more detail, a study using RACE-PCR and NGS for glioblastoma patients in comparison to healthy controls is currently undertaken in our laboratories. Furthermore, the shared immunoglobulin variants represent interesting target molecules and will be investigated in the future.

So far, the phenotype of the CD14^+^ immunoglobulin expressing macrophages (M1 or M2 category) is ambiguous and further analyses including single-cell transcriptomics in combination with immune receptor profiling are urgently needed to characterize this specific subpopulation in more detail. Moreover, investigation of the antigen specificity of the myeloid immunoglobulins will help to further uncover the role of tumor-associated macrophages in glioblastoma. Since TAM are discussed as potential targets in glioblastoma therapy [51], immunoglobulins expressed by TAM might have an impact on future therapeutic approaches.

In conclusion, the expression of variable immunoglobulins in monocytes and TAM can be a useful tool for the characterization of the tumor-infiltrating myeloid cells, as a therapeutic target or as a diagnostic marker for glioblastoma patients.

## Additional file

**Additional file 1.** Additional tables and figures.

## Abbreviations

ATRX: ATP-dependent helicase
CCL2: chemokine (C–C motif) ligand 2, also referred to as monocyte chemoattractant protein 1 (MCP1)
CCL22: chemokine (C–C motif) ligand 22, also referred to as macrophage-derived chemokine (MDC)
CCL3: chemokine (C–C motif) ligand 4, also referred to as macrophage Inflammatory protein 1-alpha (MIP1α)
CD: cluster of differentiation
CDR3: complementarity-determining region
CXCL8: C-X-C motif chemokine 8, also referred to as Interleukin 8 (IL-8)
CXCL10: C-X-C motif chemokine 10, also referred to as Interferon gamma-induced protein 10 (IP-10)
EGF: epidermal growth factor
GAPDH: glycerinaldehyd-3-phosphat-Dehydrogenase
GBM: glioblastoma multiforme
IDH: isocitrate dehydrogenase
Ig: immunoglobulin
IL-10: interleukin 10
NGS: next-generation-sequencing
PBS: phosphate-buffered saline
PCR: polymerase chain reaction
sCD40L: soluble CD40-ligand
TAM: tumor-associated macrophages
TCR: T cell receptor
TNFα: tumor-necrosis factor alpha
TIL: tumor-infiltrating lymphocytes

## References

[1] Chávez-Galán L, Olleros ML, Vesin D, Garcia I (2015). Much more than M1 and M2 macrophages, there are also CD169^+^ and TCR^+^ macrophages. Front Immunol..

[2] Janeway CA, Medzhitov R (2002). Innate immune recognition. Annu Rev Immunol.

[3] Hirano M, Das S, Guo P, Cooper MD (2011). The evolution of adaptive immunity in vertebrates. Adv Immunol.

[4] Kaminski WE, Beham AW, Puellmann K (2012). Extralymphocytic flexible immune recognition: a new angle on inflammation and aging. Aging Dis..

[5] Kaminski WE, Beham AW, Kzhyshkowska J, Gratchev A, Puellmann K (2013). On the horizon: flexible immune recognition outside lymphocytes. Immunobiol..

[6] Puellmann K, Kaminski WE, Vogel M, Nebe CT, Schroeder J, Wolf H, Beham AW (2006). From the cover: a variable immunoreceptor in a subpopulation of human neutrophils. Proc Natl Acad Sci USA.

[7] Puellmann K, Beham AW, Kaminski WE (2006). Cytokine storm and an anti-CD28 monoclonal antibody. N Engl J Med.

[8] Fuchs T, Puellmann K, Scharfenstein O (2012). The neutrophil variable TCR-like immune receptor is expressed across the entire human life span but repertoire diversity declines in old age. Biochem Biophys Res Commun.

[9] Beham AW, Puellmann K, Laird R (2011). A TNF-regulated recombinatorial macrophage immune receptor implicated in granuloma formation in tuberculosis. PLoS Pathog.

[10] Fuchs T, Puellmann K, Hahn M (2013). A second combinatorial immune receptor in monocytes/macrophages is based on the TCRγδ. Immunobiol..

[11] Legrand F, Driss V, Woerly G (2009). A functional gammadelta TCR/CD3 complex distinct from gammadelta T cells is expressed by human eosinophils. PLoS ONE.

[12] Chorazeczewski JK, Aleshnick M, Majam V (2018). TCRβ combinatorial immunoreceptor expression by neutrophils correlates with parasite burden and enhanced phagocytosis during a Plasmodium berghei ANKA malaria infection. Infect Immun.

[13] Oakley MS, Chorazeczewski JK, Aleshnick M (2018). TCRβ-expressing macrophages induced by a pathogenic murine malaria correlate with parasite burden and enhanced phagocytic activity. PLoS ONE.

[14] Fuchs T, Puellmann K, Schneider S (2012). An autoimmune double attack. Lancet.

[15] Lakschevitz FS, Aboodi GM, Glogauer M (2013). Oral neutrophils display a site-specific phenotype characterized by expression ofT-cell receptor. J Periodontol.

[16] Fuchs T, Puellmann K, Emmert A (2015). The macrophage-TCRαβ is a cholesterol-responsive combinatorial immune receptor and implicated in atherosclerosis. Biochem Biophys Res Commun.

[17] Fuchs T, Hahn M, Riabov V (2017). A combinatorial T cell receptor expressed by macrophages in the tumor microenvironment. Immunobiol..

[18] Huang J (2014). Rearrangement and expression of the immunoglobulin mu-chain gene in human myeloid cells. Cell Mol Immunol.

[19] Wang C, Xia M, Sun X (2015). IGK with conserved IGKV/IGKJ repertoire is expressed in acute myeloid leukemia and promotes leukemic cell migration. Oncotarget.

[20] Fuchs T, Hahn M, Ries L (2018). Expression of combinatorial immunoglobulins in macrophages in the tumor microenvironment. PLoS ONE.

[21] Raggi F, Pelassa S, Pierobon D, Penco F, Gattorno M, Novelli F (2017). Regulation of human macrophage M1–M2 polarization balance by hypoxia and the triggering receptor expressed on myeloid cells-1. Front Immunol..

[22] Mantovani A, Schioppa T, Porta C, Allavena P, Sica A (2006). Role of tumor-associated macrophages in tumor progression and invasion. Cancer Metastasis Rev.

[23] Lewis CE, Pollard JF (2006). Distinct role of macrophages in different tumor microenvironments. Cancer Res.

[24] van der Bij GJ, Oosterling SJ, Meijer S, Beelen RHJ, van Egmond M (2005). The role of macrophages in tumor development. Cell Oncol..

[25] Ponzoni M, Pastorino F, Di Paolo D, Perri P, Brignole C (2018). Targeting macrophages as a potential therapeutic intervention: impact on inflammatory diseases and cancer. Int J Mol Sci.

[26] Porta C, Sica A, Riboldi E (2017). Tumor-associated myeloid-cells: new understandings on their metabolic regulation and their influence in cancer immunotherapy. Febs J.

[27] Noy R, Pollard JW (2014). Tumor-associated macrophages: from mechanisms to therapy. Immunity.

[28] Hanada T, Nakagawa M, Emoto A, Nomura T, Nasu N, Nomura Y (2000). Prognostic value of tumor-associated macrophage count in human bladder cancer. Int J Urol.

[29] Ostrom QT, Gittleman H, Stetson L, Virk SM, Barnholtz-Sloan JS, Raizer J, Parsa A (2015). Epidemiology of gliomas. Current understanding and treatment of gliomas.

[30] Stupp R, Mason WP, van den Bent MJ, Weller M, Fisher B, Taphoorn MJ (2005). Radiotherapy plus concomitant and adjuvant temozolomide for glioblastoma. N Engl J Med.

[31] Wen PY, Kesari S (2008). Malignant gliomas in adults. N Engl J Med.

[32] Ohgaki H (2009). Epidemiology of brain tumors. Methods Mol Biol.

[33] Martinez R, Schackert G, Yaya-Tur R, Rojas-Marcos I, Herman JG, Esteller M (2007). Frequent hypermethylation of the DNA repair gene MGMT in long-term survivors of glioblastoma multiforme. J Neurooncol.

[34] Pearson JRD, Regad T (2017). Targeting cellular pathways in glioblastoma multiforme. Signal Transduct Target Ther..

[35] Krex D, Klink B, Hartmann C, von Deimling A, Pietsch T, Simon M (2007). Long-term survival with glioblastoma multiforme. Brain.

[36] Han Y, Li H, Guan Y, Huang J (2016). Immune repertoire: a potential biomarker and therapeutic for hepatocellular carcinoma. Cancer Lett.

[37] Shannon CE (2001). A mathematical theory of communication. ACM Sigmob Mob Comput Commun Rev..

[38] Estorninho M, Gibson VB, Kronenberg-Versteeg D, Liu Y, Ni C, Cerosaletti K, Peakman M (2013). A novel approach to tracking antigen-experienced CD4 T cells into functional compartments via tandem deep and shallow TCR clonotyping. J Immunol..

[39] Brezinschek HP, Foster SJ, Brezinschek RI, Dörner T, Domiati-Saad R, Lipsky PE (1997). Analysis of the human VH gene repertoire: differential effects of selection and somatic hypermutation on human peripheral CD5^+^/IgM^+^ and CD5-/IgM^+^ B cells. J Clin Invest..

[40] Gustafson MP, Lin Y, New KC, Bulur PA, O’Neill BP, Gastineau DA, Dietz AB (2010). Systemic immune suppression in glioblastoma: the interplay between CD14^+^HLA-DR^lo/neg^ monocytes, tumor factors, and dexamethasone. Neuro Oncol.

[41] Kumar R, Kamdar D, Madden L (2006). Th1/Th2 cytokine imbalance in meningioma, anaplastic astrocytoma and glioblastoma multiforme patients. Oncol Rep.

[42] Shadhi S, Chaudhry MN, Mahomood N, Hussain K, Sheikh S, Ahmed N (2015). Hematological profiling and assessment of suspected environmental factors of brain tumor patients: a prospective study of teriary care hospital in Pakistan. J Neurol Sci Turk.

[43] Winkler MS, Rissiek A, Priefler M, Schwedhelm E, Robbe L, Bauer A (2017). Human leukocyte antigen (HLA-DR) gene expression is reduced in sepsis and correlates with impaired TNAα response: a diagnostic tool for immunosuprresion. PLoS ONE.

[44] Patel AP, Tirosh I, Trombetta JJ (2014). Single-cell RNA-seq highlights intratumoral heterogeneity in primary glioblastoma. Science.

[45] Walentynowicz KA, Ochocka N, Pasierbinska M (2018). In search for reliable markers of glioma-induced polarization of microglia. Front Immunol.

[46] Zisakis A, Piperia C, Themistocleous MS (2007). Comparative analysis of peripheral and localised cytokine secretion in glioblastoma patients. Cytokine.

[47] Zhou M, Bracci PM, McCoy LS (2015). Serum macrophage-derived chemokine/CCL22 levels are associated with glioma risk, CD4 T cell lymphopenia and survival time. Int J Cancer.

[48] Feng L, Qian H, Yu X (2017). Heterogeneity of tumor-infiltrating lymphocytes ascribed to local immune status rather than neoantigens by multi-omics analysis of glioblastoma multiforme. Sci Rep..

[49] Mohme M, Schliffke S, Maire CL (2018). Immunophenotyping of newly diagnosed and recurrent glioblastoma defines distinct immune exhaustion profiles in peripheral and tumor-infiltrating lymphocytes. Clin Cancer Res.

[50] Chen Z, Feng X, Herting CJ (2017). Cellular and molecular identity of tumor-associated macrophages in glioblastoma. Cancer Res.

[51] Pyonteck SM, Akkari L, Schuhmacher AJ (2013). CSF-1R inhibition alters macrophage polarization and blocks glioma progression. Nat Med.

