# Supplementary material for: Circulating monocytes and tumor-associated macrophages express recombined immunoglobulins in glioblastoma patients
Source: Clin Transl Med. 2019 Jun 3;8:18. doi: 10.1186/s40169-019-0235-8 (PMC6545295; doi:10.1186/s40169-019-0235-8)

Table S1

Patient and tumor characteristics, co-morbidity and molecular diagnostics of patients (n=15) with glioblastoma included in this study

Patient ID	age (years)	gender	Histology	tumor stage (WHO grade)	tumor volume (ml)	progression free survival (days)	events (relapse=1)	co-morbidity	localisation	Ki 67 (%)	ATRX mutation	IDH mutation
GBM001	48	f	GBM	IV	11.03	168	1	wound healing deficit	frontoparietal left parasagittal (central)	15	1	0
GBM002	73	m	GBM	IV	8.67	73	1	epilepsy, AH, prostate cancer	frontal left peripheral	15	1	0
GBM003	62	m	Gliosarcoma	IV	74.98	235	1	PE, epilepsy, AH, NIDDM	parietotemporooccipital (spanning central to peripheral)	15	1	0
GBM004	74	f	GBM	IV	62.01	200	1	epilepsy, AH, CHD	occipital right (spanning central to peripheral)	25	1	0
GBM005	44	f	GBM	IV	36.60	153	1	hydrocephalus, shunt infection	corpus callosum (central)	20	1	0
GBM006	54	m	GBM	IV	50.49	343	1	CHD, PAD, epilepsy	frontal right (peripheral)	20	1	0
GBM007	64	m	GBM	IV	59.35	124	1	epilepsy, AH, NIDDM	frontal right (peripheral)	15	1	0
GBM008	55	m	GBM	IV	7.56	125	1	none	temporal right (peripheral)	40	1	0
GBM009	55	m	GBM	IV	91.34	249	1	none	temporal right (central)	10	1	0
GBM010	58	w	GBM	IV	41.19	388	1	epilepsy	frontal left (central and peripheral, 2 sites)	30	1	0
GBM011	70	m	GBM	IV	28.17			CHD, AH, epilepsy	temporooccipital right (central)	15	1	0
GBM012	43	w	GBM	IV	15.68	219	1	none	frontoparietal right (peripheral)	20	1	0
GBM013	47	w	Gliosarcoma	IV	50.48	36	1	hydrocephalus, epilepsy	corpus callosum (central)	50	1	0
GBM014	52	w	GBM	IV	3.54	235	1	Wegener's granulomatosis, AH, epilepsy	frontal left (peripheral)	12	1	0
GBM015	72	w	GBM	IV	34.35	169	1	epilepsy	frontal left ((spanning central to peripheral)	20	1	0
GBM016	65	m	GBM	IV	4.94	149	1	epilepsy, AH	parietal left (peripheral)	35	1	0
GBM017	55	m	GBM	IV	40.55	197	1	none	parietotemporooccipital left (spanning central to peripheral)	20	1	0

AH, arterial hypertension

NIDDM, non-insulin-dependent diabetes mellitus

PE, pulmonary embolism

CHD, coronary heart disease

PAD, peripheral arterial disease

Table S2

Shared immunoglobulin heavy and light chain CDR3 variants from B cells isolated from blood and tumor of 3 glioblastoma patients.

Overlaps heavy chains

CDR3	GBM003		GBM004		GBM008	
	TAM	blood	TAM	blood	TAM	blood
TAMVDVGGLLIGDY					X	X
VRELGQLVGFDF					X	X

Overlaps light chains

CDR3	GBM003		GBM004		GBM008	
	TAM	blood	TAM	blood	TAM	blood
IRVVIYLGR	X	X		X		
MQALQTPLT		X				X
QAWDSSTVV				X		X
QQHYNPPPMYT					X	X

Figure S1

Sensitivity of purity control PCR. RNA of $CD19^+$ cells was spiked into the RNA of isolated $CD14^+$ cells at different concentrations (0.1%; 0.5%; 1%; 2% and 5%) and the amplification of CD19 was performed using the standard PCR protocol.

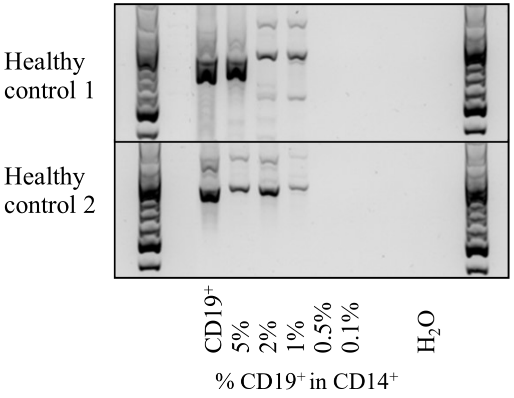

Figure S2A

Expression of the soluble and transmembrane form of IgM in $CD14^{+}$ monocytes and $CD19^{+}$ B cells of glioblastoma patients and a healthy control.

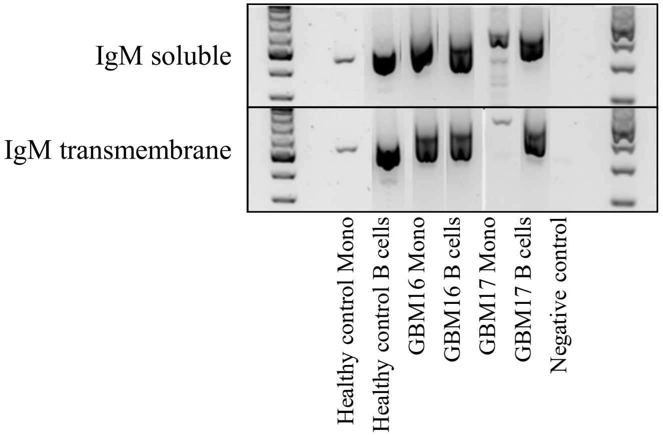

Figure S2B

IgM expression level in CD14$^+$ monocytes and CD14$^+$ TAM of six representative GBM patients. GAPDH, loading control.

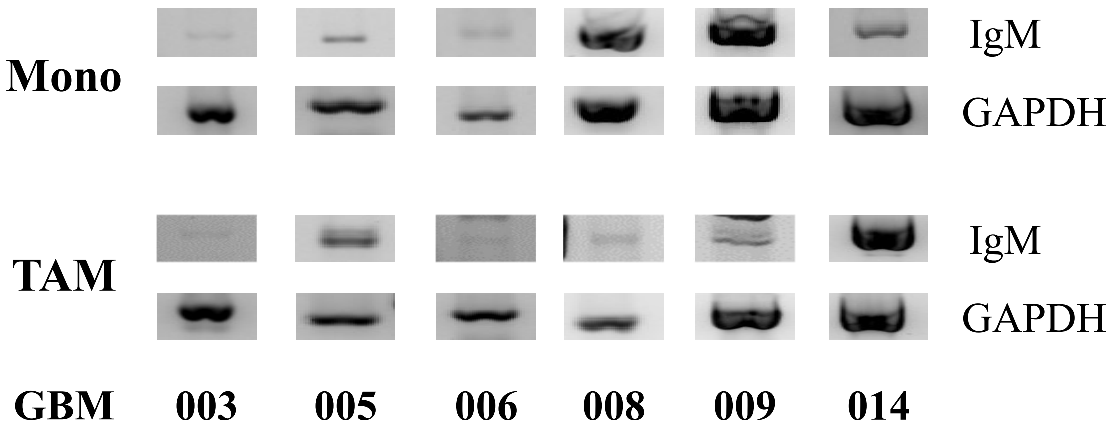

Figure S3

Shannon-Diversity-Index of expressed immunoglobulin CDR3 variants in $CD14^{+}$ and $CD19^{+}$ cells

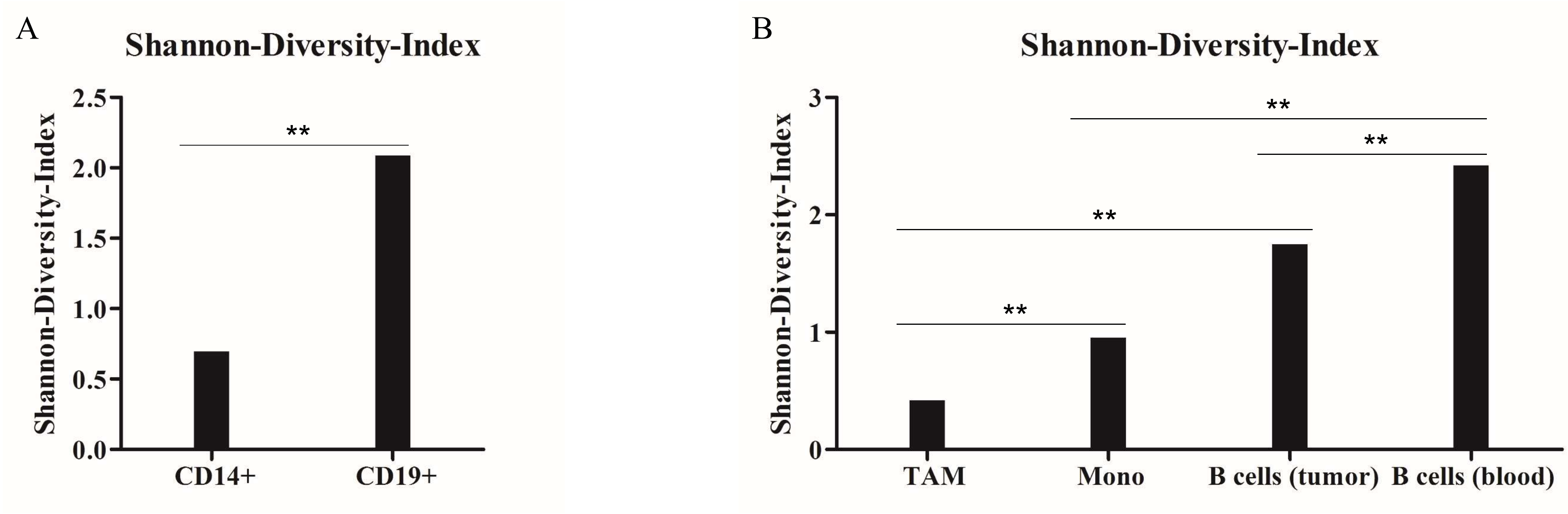

** p<0.01

Figure S4

Immunoglobulin CDR3 repertoire diversities of all 15 GBM-patients

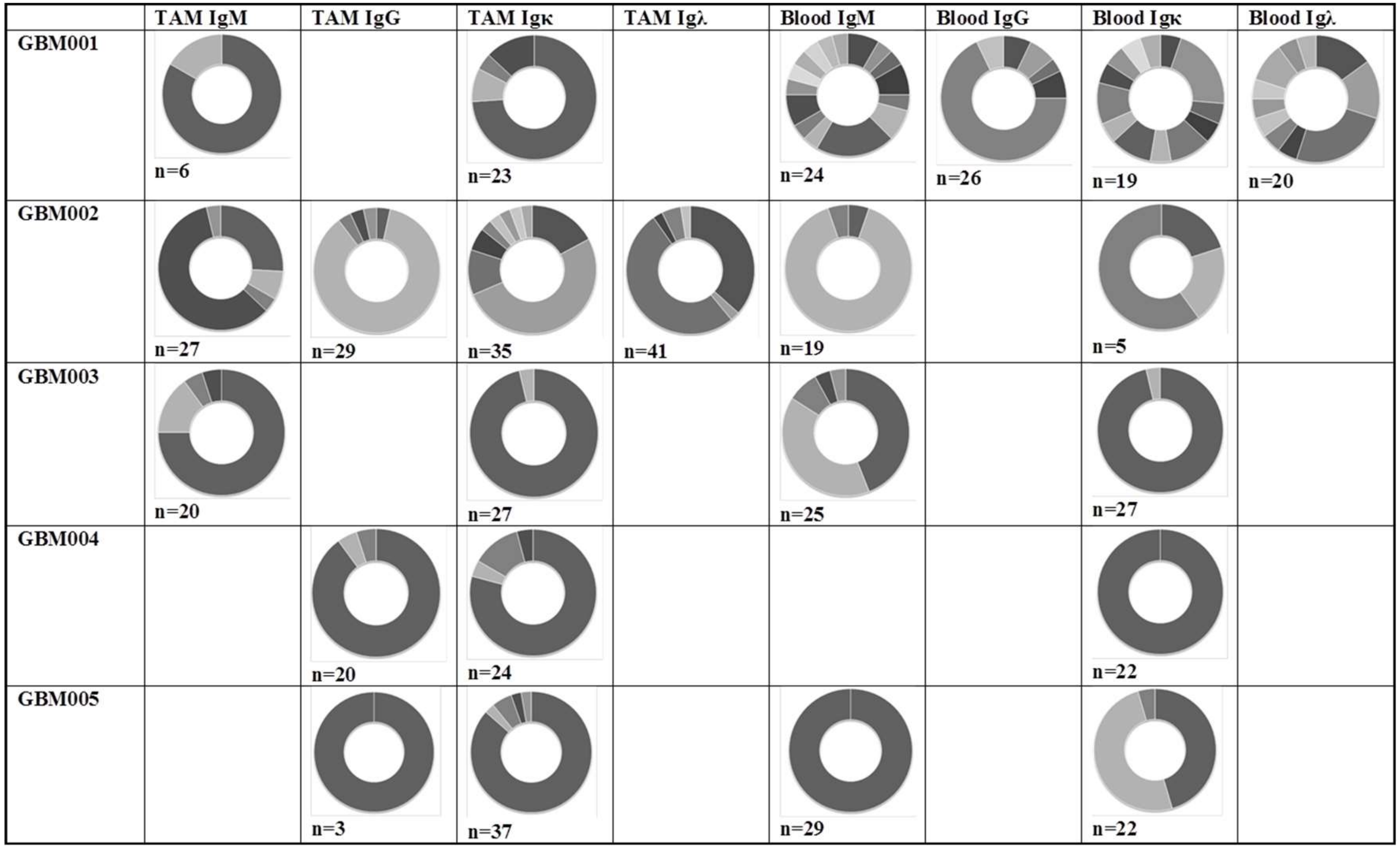

Figure S4

	TAM IgM	TAM IgG	TAM Igκ	TAM Igλ	Blood IgM	Blood IgG	Blood Igκ	Blood Igλ
**GBM006**							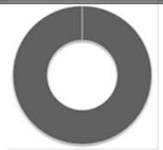 **n=10**	
**GBM007**			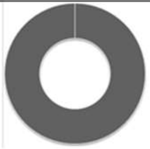 **n=2**				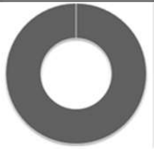 **n=7**	
**GBM008**					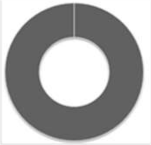 **n=3**		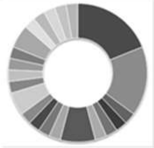 **n=37**	
**GBM009**							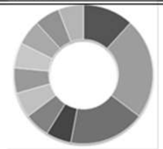 **n=17**	
**GBM010**			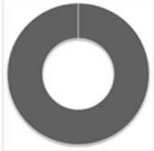 **n=3**				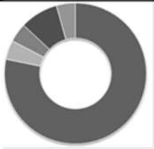 **n=23**	

Figure S4

	TAM IgM	TAM IgG	TAM Igκ	TAM Igλ	Blood IgM	Blood IgG	Blood Igκ	Blood Igλ
GBM011							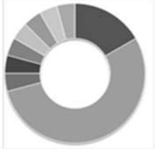<br>n=24	
GBM012	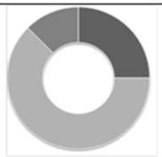<br>n=8	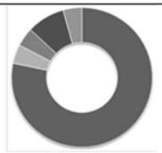<br>n=23	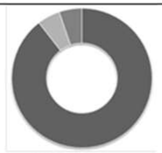<br>n=19		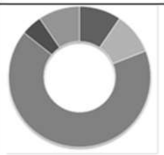<br>n=21	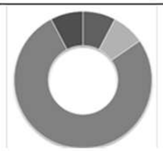<br>n=13		
GBM013	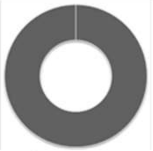<br>n=4		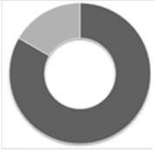<br>n=18		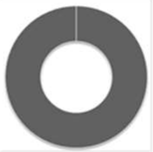<br>n=6	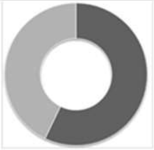<br>n=7	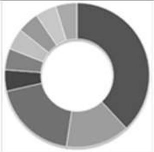<br>n=21	
GBM014							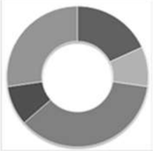<br>n=11	
GBM015					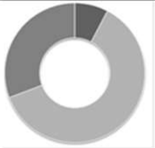<br>n=13			

Figure S5

The specific tumor volume and the number of isolated CD14^{+} cells from blood and tumor tissue of glioblastoma patients do not correlate.

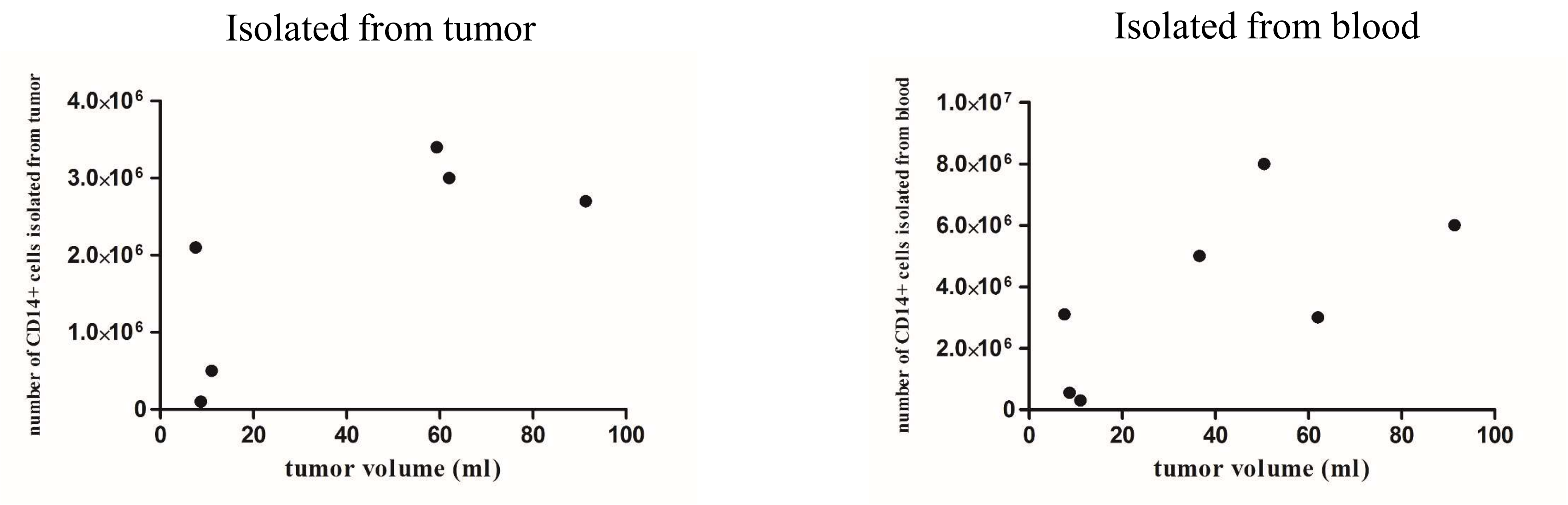

Figure S6

HE- and CD14-stainings of GBM-patients with different tumor volumes (in order of decreasing tumor volume).

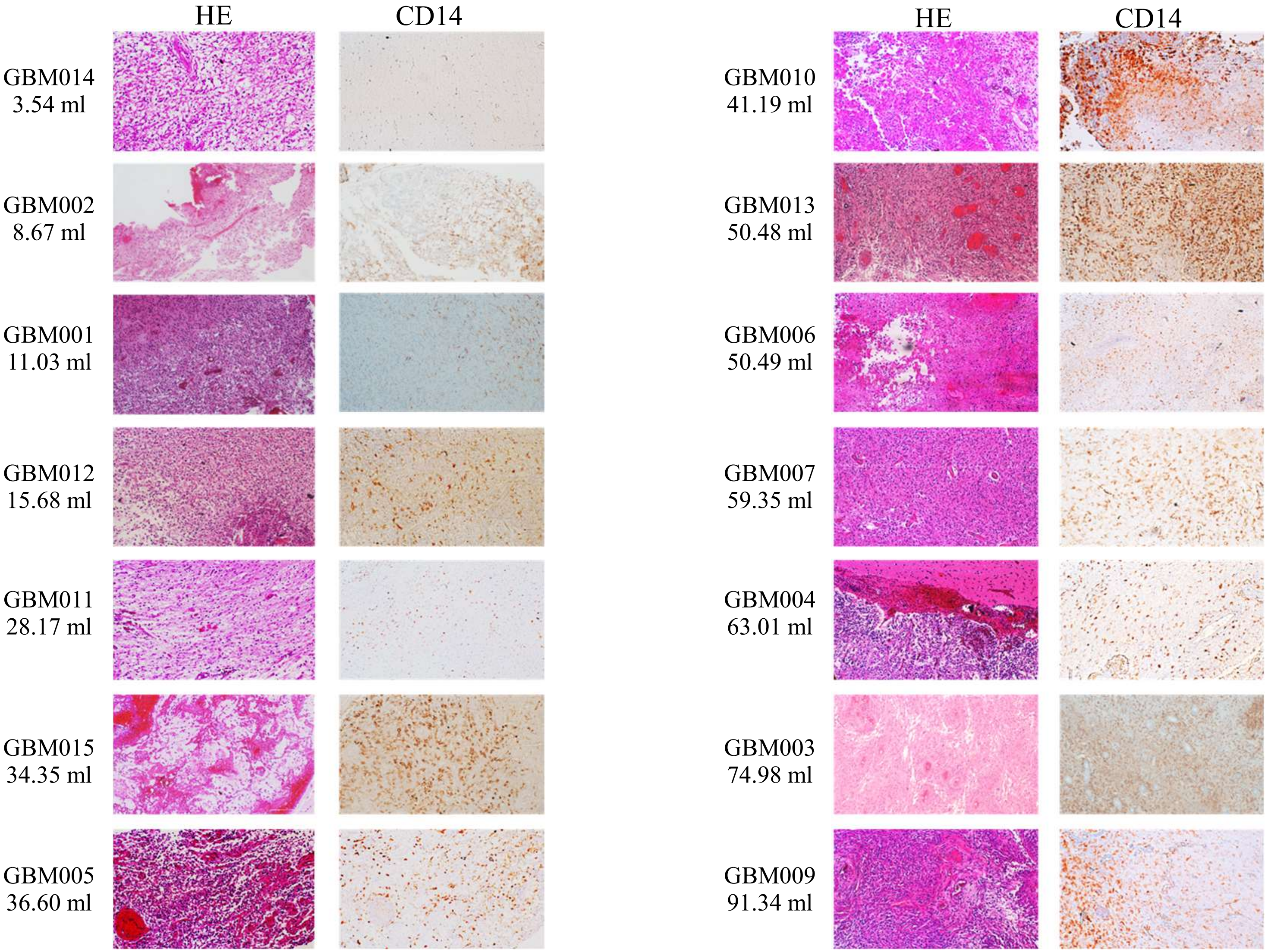

Figure S7

Distribution of the expressed immunoglobulin VH-chains on the locus of chromosome 14 (all patients).

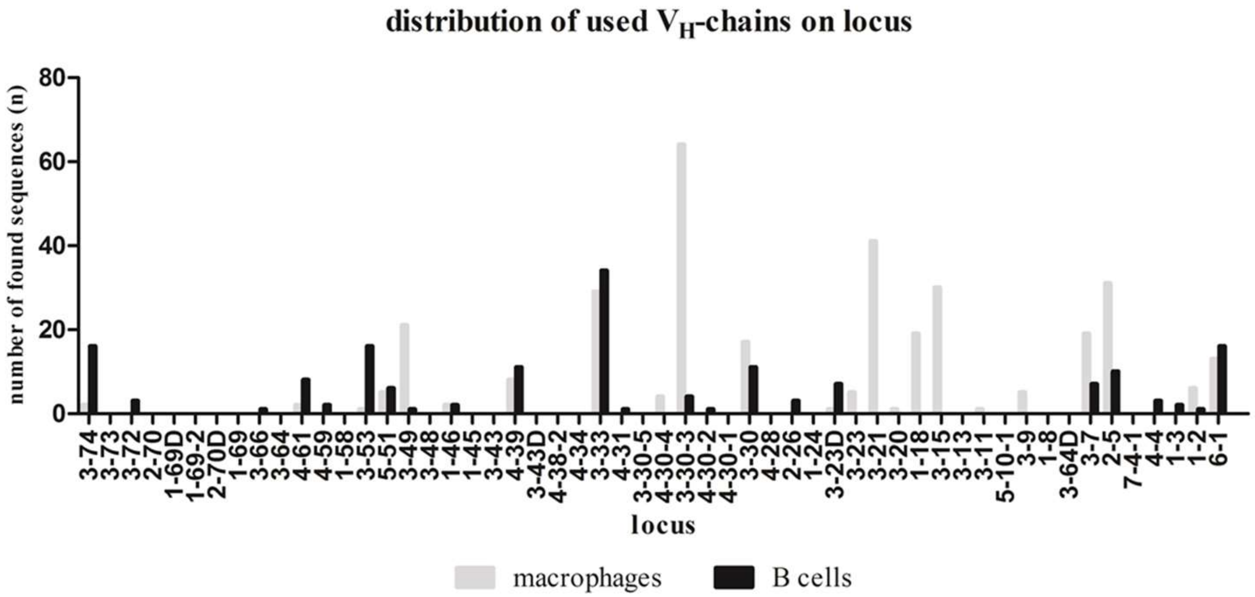

Figure S8A

Serum levels of several cytokines released from healthy controls (n=7) and GBM patients (n=11). *p<0.05; **p<0.001

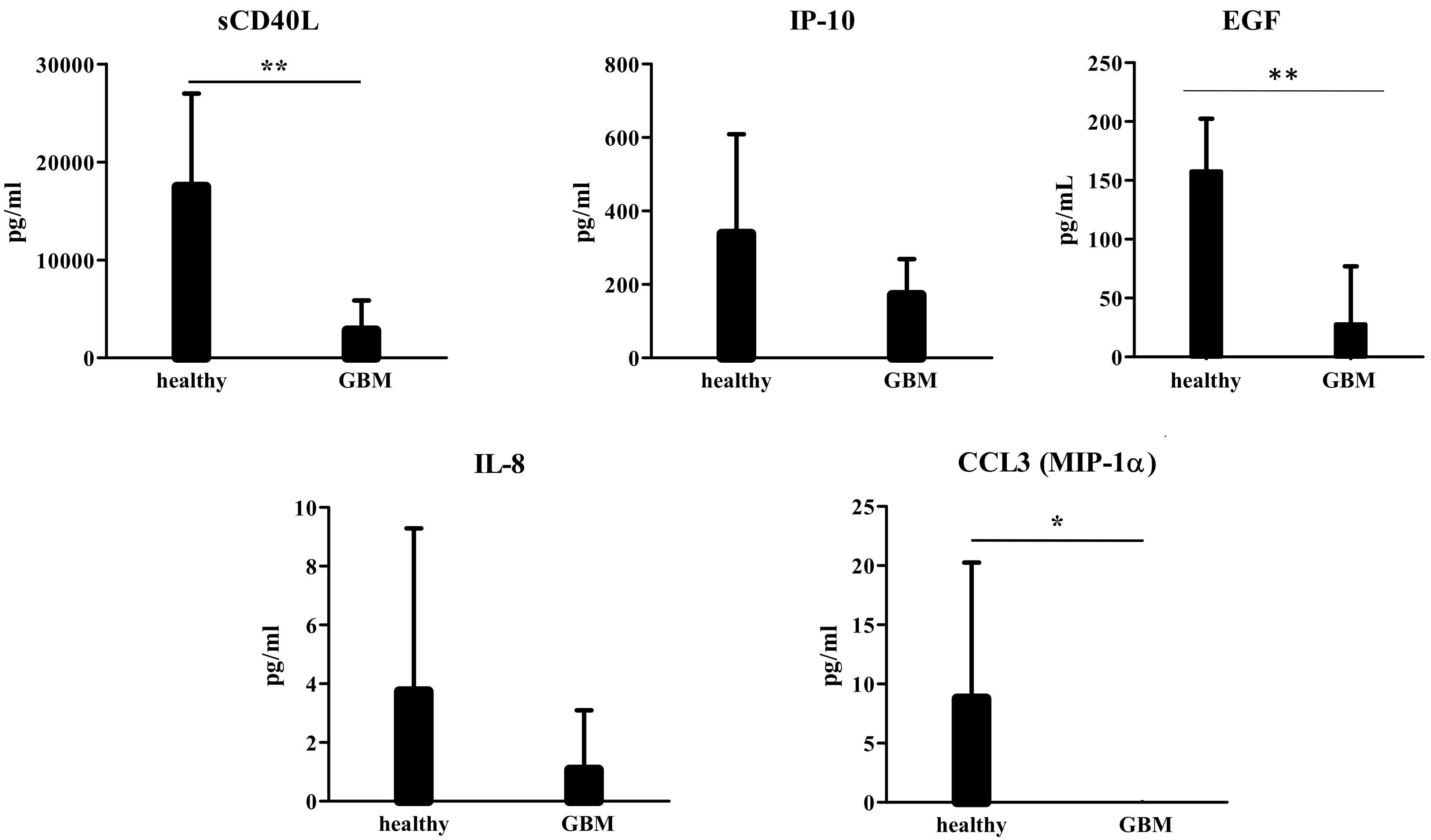

Figure S8B

Serum levels of several cytokines released from healthy controls (n=7) and GBM patients (n=11). *p<0.05; **p<0.001

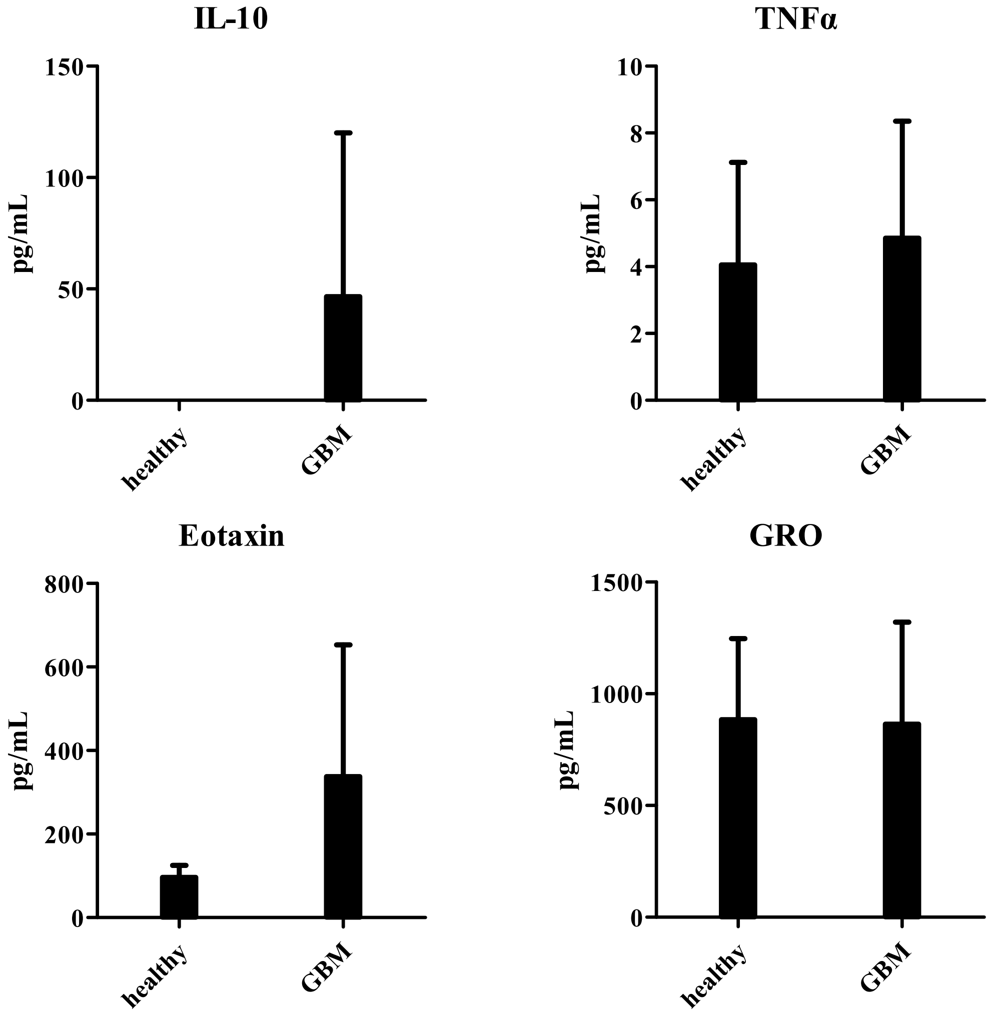

Figure S9

Diverse expression levels of HLA-DR in myeloid cells from healthy individuals and GBM patients.

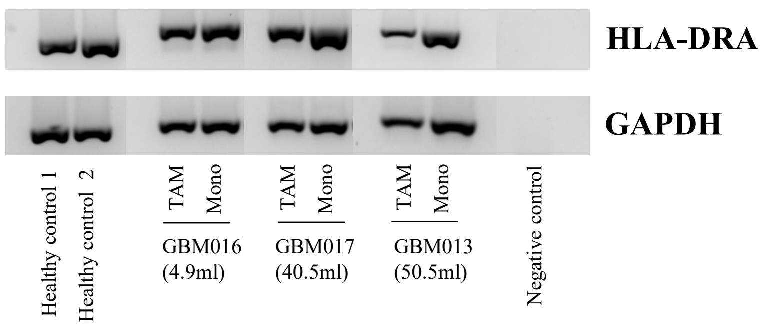

Supplement: Supplementary file 1 — Additional file 1. Additional tables and figures. [file 40169_2019_235_MOESM1_ESM.pdf]
